# Characterisation of the Faecal Microbiota in Dogs with Mast Cell Tumours Compared with Healthy Dogs

**DOI:** 10.3390/ani15152208

**Published:** 2025-07-27

**Authors:** Catarina Aluai-Cunha, Diana Oliveira, Hugo Gregório, Gonçalo Petrucci, Alexandra Correia, Cláudia Serra, Andreia Santos

**Affiliations:** 1 Department of Veterinary Clinics, Institute of Biomedical Sciences Abel Salazar (ICBAS), University of Porto, R. Jorge Viterbo Ferreira 228, 4050-313 Porto, Portugal; dianaifo@gmail.com (D.O.); aasantos@icbas.up.pt (A.S.); 2 Animal Science and Study Centre (CECA) 4050-313 Porto, Portugal; 3 Institute for Research and Innovation in Health (I3S), University of Porto, R. Alfredo Allen 208, 4200-135 Porto, Portugal; iacorreia@icbas.up.pt; 4 Interdisciplinary Centre of Marine and Environmental Research (CIMAR/CIIMAR), Av. General Norton de Matos S/N, 4450-208 Matosinhos, Portugal; claudia.serra@fc.up.pt; 5 Anicura CHV—Veterinary Hospital Center, R. Manuel Pinto de Azevedo 118, 4100-320 Porto, Portugal; hugo.gregorio@anicura.pt; 6 Animal and Veterinary Department, University Institute of Health Sciences, IUCS-CESPU, Rua Central de Gandra, 1317, 4585-166 Gandra PRD, Portugal; goncalo.petrucci@onevetgroup.pt; 7 Onevetgroup Hospital Veterinário do Porto (HVP), 4150-562 Porto, Portugal; 8 Animal and Veterinary Research Centre (CECAV), University of Trás-os-Montes and Alto Douro, 5000-801 Vila Real, Portugal; 9 Department of Immuno-Physiology and Pharmacology, Institute of Biomedical Sciences Abel Salazar (ICBAS), University of Porto, R. Jorge Viterbo Ferreira 228, 4050-313 Porto, Portugal; 10 Department of Biology, Faculty of Sciences (FCUP), University of Porto, Rua do Campo Alegre S/N, 4169-007 Porto, Portugal

**Keywords:** dog, dysbiosis, mast cell tumours, microbiota

## Abstract

Mast cell tumours (MCT) are the most common cutaneous neoplasms in dogs, with variable behaviours and patient survival time. Both indolent and aggressive forms have been described, but much remains to be explored regarding prognosis and therapy. Evidence has highlighted the influence of microbiota on multiple health and disease processes, including certain types of cancer in humans. However, knowledge remains scarce regarding microbiota biology and its interactions in both humans and canine cancer patients. This study aimed to characterise the faecal microbiota of dogs with MCT and compare it with that of healthy individuals. Twenty-eight dogs diagnosed with MCT and twenty-eight healthy dogs were enrolled in the study. Faecal samples were collected and analysed by Illumina sequencing of 16S rRNA genes. Alpha diversity was significantly lower in dogs with cancer, and the species diversity InvSimpson Indexwas reduced (*p* = 0.019). Principal coordinate analysis showed significant differences in the bacterial profile of the two groups: there was a significant lower abundance of the genera *Alloprevotella*, *Holdemanella*, *Erysipelotrichaceae_UCG-003*, and *Anaerobiospirillum* and, conversely, a significant increase in the genera *Escherichia-Shigella* and *Clostridium sensu stricto 1* in diseased dogs. At the phylum level, *Bacteroidota* was significantly reduced in diseased dogs (25% in controls vs. 19% in MCT dogs). In conclusion, sequencing analysis provided an overview of the bacterial profile and showed statistical differences in the microbial communities of dogs with MCT compared with healthy dogs, suggesting a link between the gut microbiota and MCT in this species.

## Simple Summary

Studying the relationship between microbiome and oncological diseases in dogs is crucial, as emerging evidence suggests that microbial communities play a significant role in cancer development, progression, and response to treatment. By exploring how the microbiome influences the canine immune system and tumour microenvironment, researchers can identify novel biomarkers for early diagnosis and prognosis, as well as develop more targeted and personalised therapeutic strategies. This field not only has the potential to improve cancer outcomes in veterinary medicine but may also provide valuable comparative insights for human oncology. Mast cell tumours are among the most relevant diseases in dogs and, in this study, we found statistical differences in the faecal microbiota profile between healthy and diseased dogs, suggesting that the microbiome may play a role in the development or progression of this disease.

## 1. Introduction

The prevalence of oncological diseases is increasing in dogs, representing 30% of canine causes of death [1]. In this species, mast cell tumours (MCT) are the most frequent malignant skin neoplasms. Dogs with high-grade neoplasms have a poor prognosis, with short survival times and limited treatment options [2]. In both human and canine species, MCT exhibiting KIT (proto-oncogene receptor tyrosine kinase) mutations appear to contribute to the evolution of the disease, encouraging the inclusion of tyrosine kinase inhibitors in treatment. However, clinical responses are often transient, and research for more effective therapies is needed [2]. Evidence has highlighted the influence of the faecal microbiota in multiple disease processes [3], and a line of research gaining attention in humans is the microbiota–cancer interaction.

The human and animal microbiota are composed of bacteria, fungi, protozoa, and viruses that cohabit the organism. Their genes and interactions collectively define the term microbiome. The microbiome balance can be altered, which deregulates genetic and metabolic pathways as well as immunological responses, leading to diseases such as cancer [4]. Reduced stability of microbial communities [5], a decrease in overall bacterial variety (the alpha diversity) [6], and a reduction in obligate anaerobes from the phyla *Firmicutes* and *Bacteroidota*, followed by an increase in facultative anaerobes—including members of the family *Enterobacteriaceae*—have been described as major hallmarks of dysbiosis in different species [5,6,7,8]. The microbiota metabolises undigested substrates, desquamated epithelial cells, and endogenous mucus that pass through the small intestine. Saccharolytic fermentation of carbohydrates escaping digestion and absorption in the small intestine produces short-chain fatty acids (SCFA), which supply energy for bacterial metabolism and epithelial cell growth [9]. The major products are formate, acetate, propionate, and butyrate. The interaction between the immune system and commensal microorganisms is an essential part of physiological homeostasis in both health and disease. Emerging studies suggest that altered immunological responses to commensal microorganisms can cause chronic inflammation and oncological diseases [10]. Notable examples include *Fusobacterium nucleatum* and *Bacteroides fragilis* in the colon. These species produce virulence factors such as VacA (vacuolating cytotoxin A), urease, CagA (cytotoxin-associated gene A), and NapA2 (neutrophil-activating protein A), which contribute to chronic inflammation and host DNA damage, leading to carcinogenesis and tumour progression [9]. In colorectal cancer, *Fusobacterium nucleatum* secretes Fap2 protein, which invades host cells by binding to the Gal-GalNAc polysaccharide and interacting with E-cadherin. This interaction activates β-catenin signalling and induces the overexpression of NF-κB, ERK, and STAT pathways, thereby modulating immune cell recruitment and proliferation, enhancing cell survival and migration, and facilitating tumour development [9]. Several reports have revealed the contribution of changes in the microbiota to the development of human cancers such as colorectal [11], prostate [12], and pancreatic cancer [13]. In breast cancer patients, altered tumour and gut microbiota populations have been shown to compromise responses to chemotherapeutic protocols and immunotherapy [14]. The modulation of the microbiota with probiotics has also demonstrated therapeutic benefits [14].

In 2022, a new version of one of the most important articles in the field of oncology—*Hallmarks of cancer*: *New Dimensions* [15]—was published, in which the microbiome is now recognised as a fundamental factor in the development of oncological diseases. Research in this field is highly relevant in veterinary medicine to better understand the relationship between microbes and disease. However, there are only a few reports characterising the faecal microbiota in animals with cancer—namely in feline alimentary small cell lymphoma [16], and in dogs with multicentric [17] and intestinal lymphoma [18], colorectal epithelial tumours [19], and in mammary tumours [20].

Concerning MCTs, there is only one study that characterised the skin surface and dermal microbiota of 11 affected dogs, using contralateral skin sites as intra-animal healthy controls [21]. The authors found that the microbial profile differed between healthy and tumour-affected skin and dermis, indicating that changes in microbiota composition are linked to the disease. Taxonomy analysis revealed an increased abundance of the family *Corynebacteriaceae* and the phylum *Firmicutes* on the MCT-affected skin surface compared to healthy tissues [21]. To date, there are no studies exploring the role of the faecal microbiota in the carcinogenesis of MCTs in either humans or dogs. Integrative microbiome studies may help clarify the carcinogenesis process and contribute to the development of new diagnostic, prognostic, and therapeutic biomarkers for both veterinary and human oncology.

## 2. Materials and Methods

All procedures were carried out in accordance with ethical approval from the Organization Responsible for Ethics and Animal Well Being of the ICBAS—University of Porto (367/2020/ORBEA). Only dogs whose owners signed the informed consent were enrolled in the study. Participants were selected and separated into two groups. The control group consisted of 28 dogs that had routine check-ups at the Veterinary Hospital of the University of Porto—UPVet. All canine controls were healthy at the time of sample collection, and the evaluations were conducted by two licensed veterinarians: anamnesis and physical examination (body condition score, temperature, mucous membrane inspection, hydration status, auscultation, pulse evaluation, and lymph node and abdominal palpation), as well as behavioural observation to detect any signs of systemic illness or discomfort. Complete blood work confirmed their healthy status, and none of the dogs presented gastrointestinal signs or received antibiotics within at least the previous month before sample collection. Faecal samples were also examined macroscopically for consistency, colour, and the presence of abnormalities such as mucus, blood, or parasites. All tutors completed a questionnaire assessing the health status of the animals and the patients’ medical histories were confirmed. The experimental group consisted of 28 dogs diagnosed by cytology and histopathology with MCT, attending Porto Veterinary Hospitals. All dogs were clinically staged according to World Health Organization (WHO) criteria for MCT staging before treatment, underwent abdominal ultrasound, hepato-splenic and lymph node cytology, and were treated according to the clinical oncologist’s recommendations, following owner consent. Exclusion criteria included: more than one tumour subtype at presentation; previous use of cancer medications; antibiotics and/or pre/probiotics administered within at least one month prior to sample collection; and the presence of non-neoplastic concomitant diseases.

Fresh faecal samples from both groups were collected by rectal exam under aseptic conditions and immediately stored at −80 °C until analysis, before the beginning of treatments. Genomic DNA from faecal samples was extracted from 250 mg (wet weight), using a bead-beating method and a DNA isolation kit (PowerSoil, Quiagen, Hilden, Germany), following the manufacturer’s instructions. The extracted DNA were used for library construction for Illumina sequencing, targeting the hypervariable V3–V4 region of the 16S rRNA gene. The first PCR reactions were performed for each sample using the KAPA HiFi HotStart PCR Kit (Kapa Biosystems Inc., Wilmington, MA, USA), according to the manufacturer’s suggestions, containing 0.3 μM of each PCR primer—forward primer Bakt_341F 5′–CCTACGGGNGGCWGCAG-3′ and reverse primer Bakt_805R 5′–GACTACHVGGGTATCTAATCC-3′ [22,23]—and 2.5 μL of template DNA in a total volume of 25 μL. The PCR conditions involved a 3 min denaturation at 95 °C, followed by 30 cycles of 98 °C for 20 s, 55 °C for 30 s, and 72 °C for 30 s, and a final extension at 72 °C for 5 min. In the second PCR reaction, index and sequencing adapters were added to both ends of the amplified target region, according to the manufacturer’s recommendations. Negative PCR controls were included for all amplification procedures. PCR products were then purified in one step and normalised using SequalPrep 96-well plate kit (ThermoFisher Scientific, Waltham, MA, USA), pooled, and paired-end sequenced in the Illumina MiSeq^®^ sequencer with the Miseq Reagent Kit v3 (600 cycles), according to manufacturer’s instructions (Illumina, San Diego, CA, USA), at Genoinseq (Cantanhede, Portugal). Raw reads were extracted from Illumina MiSeq^®^ system in FASTQ format. The QIIME2 package (version 2022.11) was used for ASV (amplicon sequence variants) generation and taxonomic identification. Denoising was carried out using DADA2 (v1.22.0) [24], which detected and corrected sequencing errors, removed chimeric sequences based on the consensus method, and filtered out phiX reads. After denoising, taxonomic assignments were determined for ASVs using the q2 feature-classifier plugin against the SILVA database (version 138.1) [25].

Sample coverage, alpha-diversity metrics, Bray–Curtis dissimilarity, and non-metric multidimensional scaling (NMDS) were calculated using the phyloseq R package. Ellipses were drawn on NMDS plots using vegan’s CovEllipse function. DESeq2 with the Wilcoxon hypothesis test, implemented in the phyloseq R package, was also used to determine significant differences in ASV, genus, and family abundances between healthy and diseased dogs. Only ASVs with a total read count ≥ 10 were considered for comparison in DESeq2 analyses. All statistical analyses on microbiome data were carried out using R (R 3.6.2) software. Data visualization and further statistical analysis were performed by using STAMP (v2.1.3) and R software. White’s non-parametric *t*-test was used to evaluate differences in the relative abundances of bacterial taxa between healthy dogs and those with MCT. A value of *p* ≤ 0.05 was considered statistically significant for all analyses.

## 3. Results

The clinical and clinicopathological findings of the dogs enrolled in the study are summarised in Table 1 and Table 2. All 28 dogs with MCT presented with cutaneous or subcutaneous masses and showed no or only mild alterations in biochemical profiles and haematological analysis. Twenty-four animals underwent surgical excision as first-line treatment and four animals underwent chemotherapy. Following surgical excision, five masses were classified as subcutaneous, while nineteen were cutaneous and classified using the Patnaik and Kiupel histopathological grading system. The control group comprised 17 females and 11 males, with a mean age of 4.6 years. The experimental group comprised 12 females and 16 males, with a mean age of 8.9 years. It is important to emphasise that no statistically significant differences were found when comparing the microbial profile between males and females.

**Figure 1:**
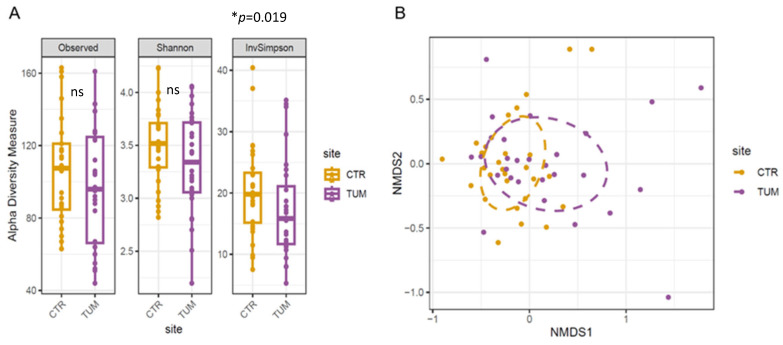
(**A**) Boxplots of alpha diversity metrics (number of Observed ASVs, Shannon Index, and Inverse Simpson Index) of the faecal material in healthy and diseased dogs. Each symbol represents one sample (n = 56, paired samples from 28 healthy and 28 diseased animals). The upper and lower edges of the boxes indicate the first and third quartiles; the line inside the box is the second quartile (median), and individual dots are outliers. * *p* = 0.019; ns = non-significant paired Wilcoxon test. (**B**) Ordination plot based on non-metric multidimensional scaling analysis of Bray–Curtis distances at the ASV level. Dots represent individual samples, and SD ellipses are coloured by sample group (healthy and diseased animals). Stress = 0.03 (stress values ≤ 0.1 are considered fair; values ≤ 0.05 indicate a good fit).

A total of 1672 ASVs (amplicon sequence variants) were obtained from sequencing the faecal samples of both healthy and diseased dogs (Supplementary Table S1). The alpha diversity analysis indicated no alterations in species richness (observed ASVs); however, the species diversity, as measured by the InvSimpson Index, was significantly reduced (*p* = 0.019) in patients with MCT compared with healthy animals (Figure 1A). The InvSimpson Index is a widely used alpha diversity metric in microbial ecology to assess diversity within a microbial community. This transformation has two important implications: the index increases with greater species richness and evenness, and it gives more weight to the most abundant species, thus emphasising the dominance structure of the community. A higher InvSimpson value indicates a more diverse microbiome—meaning not only a high number of different microbial taxa, but also that these taxa are relatively evenly distributed rather than dominated by a few species. This metric is particularly useful when comparing microbial communities because it captures changes in both the presence and relative abundance of microbes, which can be critical for understanding ecological balance, dysbiosis, or shifts due to environmental or clinical factors. The NMDS analysis revealed a more dispersed and heterogeneous distribution of the microbiota in animals with MCT, while the control animals showed a more homogeneous composition (Figure 1B).

**Figure 2:**
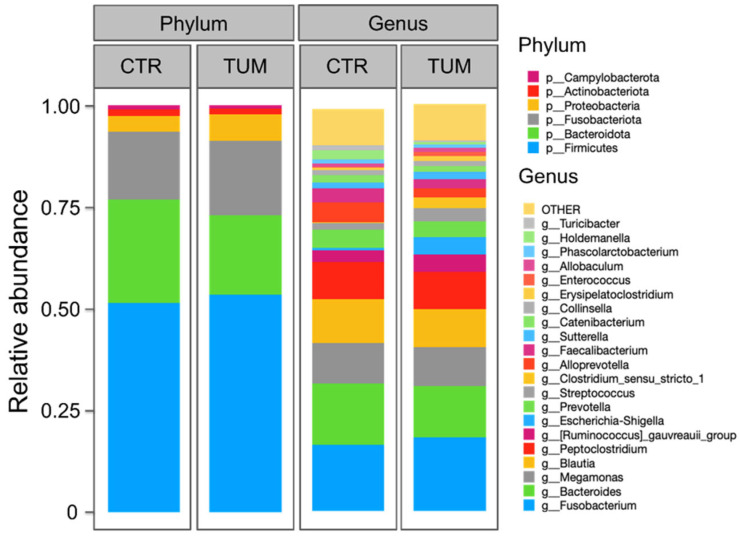
Relative bacterial abundance (*y*-axis) at the phylum and genus taxonomic levels in the faecal samples of the 28 control dogs (CTR) and 28 dogs with MCT (TUM).

**Figure 3:**
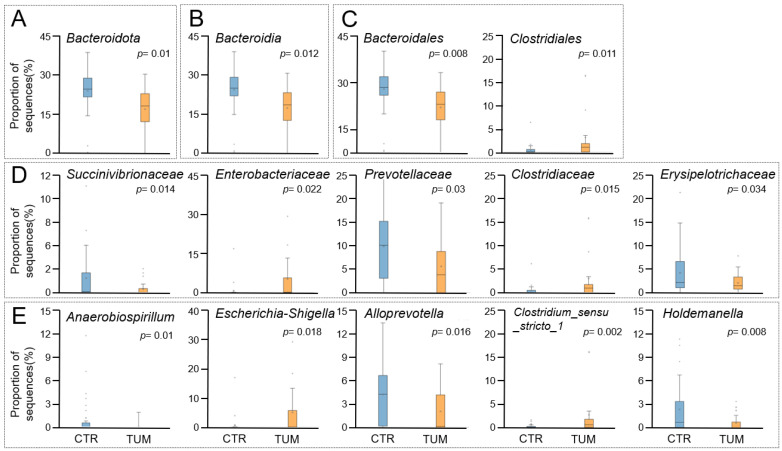
Relative proportion of sequences (*y*-axis) derived from the NGS data of taxa at the phylum (**A**), class (**B**), order (**C**), family (**D**), and genus (**E**) levels that are significantly different between faecal samples of 28 healthy (CTR) and 28 diseased dogs (TUM). Only taxa with a relative abundance higher than 1% are represented. *p*-values for each taxon are presented inside the respective graph.

The predominant bacterial phyla in faecal samples from both healthy and diseased dogs were *Firmicutes*, followed by *Bacteroidota*, *Fusobacteriota*, *Proteobacteria*, *Actinobacteriota*, and *Campylobacterota* (Figure 2). In absolute values, *Firmicutes*, *Bacteroidota*, *Actinobacteriota*, and *Campylobacterota* were more abundant in healthy animals, while *Fusobacteriota* and *Proteobacteria* were more abundant in animals with cancer (Figure 2). Despite these differences, among phyla with abundance >1%, only *Bacteroidota* (*p* = 0.01) was significantly reduced in the diseased animals (Figure 3, Supplementary Table S3).

The relative abundance of bacterial taxa at phylum, class, order, family, and genus levels with significant changes between faecal samples of healthy and diseased dogs are summarised in Figure 3 and Supplementary Table S3. Among classes with abundance >1%, only *Bacteroidia* was significantly reduced in diseased dogs (*p* = 0.012). At the order level, *Bacteroidales* were significantly lower (*p* = 0.008), whereas *Clostridiales* were significantly higher (*p* = 0.011) in diseased dogs. Among families with abundance >1%, *Clostridiaceae* (*p* = 0.015) and *Enterobacteriaceae* (*p* = 0.022) were significantly increased in animals with cancer, while *Succinivibrionaceae* (*p* = 0.014), *Erysipelotrichaceae* (*p* = 0.034), and *Prevotellaceae* (*p* = 0.030) were significantly reduced in these animals (Figure 3 and Supplementary Table S3).

The ten most abundant genera in healthy animals were *Fusobacterium*, followed by *Bacteroides*, *Blautia*, *Megamonas*, *Peptoclostridium*, *Alloprevotella*, *Prevotella*, *Faecalibacterium*, *Ruminococcus gauvreauii*, and *Holdemanella*. (Figure 2 and Supplementary Table S2). In animals with MCT, the most prevalent genera were *Fusobacterium*, followed by *Bacteroides*, *Megamonas*, *Blautia*, *Peptoclostridium*, *Ruminococcus gauvreauii*, *Escherichia-Shigella*, *Prevotella*, *Streptococcus*, and *Clostridium sensu stricto 1* (Figure 2 and Supplementary Table S2). The abundance of the genera *Alloprevotella* (*p* = 0.016), *Anaerobiospirillum* (*p* = 0.01), and *Holdemanella* (*p* = 0.008) was significantly increased in healthy animals, while the abundance of *Escherichia-Shigella* (*p* = 0.018) and *Clostridium sensu stricto 1* (*p* = 0.002) was significantly higher in diseased animals (Figure 3 and Supplementary Table S3).

**Figure 4:**
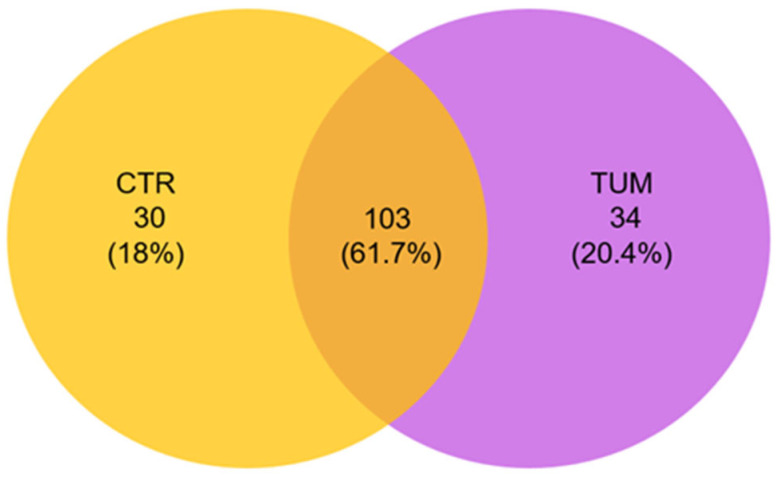
Venn diagram representation of shared and unique genera across the two groups of the study, generated using Venny URL (accessed on 16 October 2024): https://bioinfogp.cnb.csic.es/tools/venny/.

A total of 167 genera were identified in this study: A total of 103 common genera were present in the samples of both healthy and diseased animals, 30 genera were found exclusively in the healthy group, and 34 genera exclusively in the cancer group (Figure 4 and Supplementary Table S4). The 34 genera exclusively associated with animals in the cancer group were *Epulopiscium*, *Anaerococcus*, *Dialister*, *Barnesiella*, *Paeniclostridium*, *Akkermansia*, *Wolinella*, *Enterobacter*, *Bacteroidales*, *F082*, *Fastidiosipila*, *Bacillus*, *Macrococcus*, *Pasteurella*, *Pediococcus*, *Sphaerochaeta*, *Paludicola*, *Dojkabacteria*, *Haemophilus*, *Anaerostignum*, *Ploimida*, *Succiniclasticum*, *Abiotrophia*, *Tannerella*, *Treponema*, *Filifactor*, *Methanomassiliicoccus*, *Leucobacter*, *Solobacterium*, *Babeliales*, *Proteocatella*, *Aminicenantales*, *Enhydrobacter*, and *Tetrasphaera*.

## 4. Discussion

In this study, the multivariate analysis based on 16S rRNA gene sequencing provided an overview of faecal microbial communities and showed that microbiota composition diverged between healthy control dogs and dogs with MCT. As in other studies, the canine gut bacterial populations identified here belonged mainly to five phyla: *Firmicutes*, *Fusobacteria*, *Bacteroidota*, *Proteobacteria*, and *Actinobacteria* [26], with *Firmicutes* being dominant. In our study, we did not address microbiota distribution along different gastrointestinal tract segments, but rather focused on the overall community shift between healthy and MCT-affected dogs.

At the phylum level, only *Bacteroidota* was significantly reduced in the diseased animals; however, differences in abundance were also observed among other phyla. *Firmicutes*, *Actinobacteriota*, and *Campylobacterota* were more abundant in healthy animals, while *Fusobacteriota* and *Proteobacteria* were increased in animals with cancer. The reduction in *Bacteroidota* may indicate dysbiosis in MCT-affected dogs, as this phylum includes *Prevotella* and *Bacteroides*—genera considered beneficial for maintaining healthy gut homeostasis and commonly found in the faeces of healthy animals [27]. Although *Fusobacteriota* are relatively abundant in healthy canine faeces—sometimes co-dominant with *Firmicutes* and *Bacteroidota* [28]—their increase has also been associated with colorectal cancer in humans [29]. In our study, *Fusobacteriota*, particularly the related genus *Fusobacterium*, was increased in the diseased animals and appeared to be a key driver of microbiota differences between the two groups.

The dysbiosis process differs depending on the individual and the pathologic condition, but increases in facultative anaerobes, including pathogens belonging to *Escherichia*, *Shigella*, *Klebsiella*, *Salmonella*, and *Proteus* genera, are commonly described as features of dysbiosis in laboratory animals and humans [30]. Several sequencing-based studies have identified a lower relative abundance of the phylum *Bacteroidota* and increased levels of the class *Clostridia* and phylum *Proteobacteria* in dogs with dysbiosis. These studies also reported the expansion of other microorganisms that are minor constituents of the microbiota in healthy animals, as observed in our microbiota analysis results [7]. In fact, alpha diversity was significantly lower in diseased dogs than in healthy dogs, and this reduction is a hallmark of dysbiosis. The trend in our cohort mirrors common patterns of faecal dysbiosis, i.e., such as reduced bacterial diversity [6,7,8].

At the genus level, we found significant reductions in *Alloprevotella*, *Holdemanella*, *Erysipelotrichaceae_UCG-003* and *Anaerobiospirillum* in cancer dogs. *Alloprevotella* is an important SCFA producer that improves intestinal barrier function through mechanisms such as G-protein-coupled receptor-mediated inflammasome sensitisation of intestinal epithelial cells (IECs), reducing IEC oxygen concentrations and inducing hypoxia-inducible factors [31]. SCFA also exert anti-inflammatory and tolerogenic effects on immune cells. The genus *Holdemanella* has been associated with low cell proliferation of neoplastic cells in humans, due to its production of SCFA—particularly butyrate, which helps control tumour cell proliferation and protein acetylation by inhibition of calcineurin/NFATc3 activation [32]. Butyrate is produced from acetate, lactate, amino acids, and various carbohydrates via glycolysis from two different pathways: the butyryl-CoA:acetate CoA-transferase or the phosphotransbutyrykase–butyrate kinase pathway [33]. In addition to *Holdemanella*, Flint et al. [34] reported that specific families within the *Clostridiales* order (*Firmicutes*) can produce butyrate, including *Ruminococcaceae* (*Faecalibacterium*, *Subdoligranulum*), *Lachnospiraceae* (*Coprococcus*, *Eubacterium*, *Anaerostipes*, *Roseburia*), and *Erysipelotrichaceae* (*Holdemanella* family). Butyrate favours the growth of normal epithelial cells and inhibits the proliferation of tumour cells due to metabolic differences between healthy and cancerous cells. As observed in patients with colorectal cancer, tumour cells exhibit increased glycolysis instead of mitochondrial oxidative metabolism due to the Warburg effect. Consequently, they do not utilise butyrate for growth, leading to an accumulation of butyrate, which can act as a histone deacetylase inhibitor (HDACi), thereby inhibiting cell proliferation [35].

The *Erysipelotrichaceae* bacterial family is generally associated with gut health in humans [17], largely due to its production of butyrate. In our study, its abundance was higher in healthy animals compared to dogs with MCT. Zhao et al. [36] found similar results in patients with lung cancer, where this family was more abundant in the faeces of healthy individuals. In our study, *Erysipelotrichaceae_UCG-003*, an abundant genus in the healthy cohort, was the primary contributor to this increase. The *Erysipelotrichaceae* family has also been described to have a protective effect in human colorectal cancer [37]. The genus *Anaerobiospirillum* is also more abundant in the faecal microbiota population of healthy dogs and cats [38], although its abundance can vary with diet.

Our results showed that the genus *Escherichia-Shigella* was significantly increased in the faecal microbiota of dogs with cancer. Scientific literature supports these findings, reporting its increase in the faeces of humans with colorectal [39], hepatocellular [40], and pancreatic cancers [41], and in dogs with mammary tumours [20]. *Clostridium sensu stricto 1* was also significantly increased in animals with MCT. This genus is amplified in humans with urogenital cancer [42], myeloid leukaemia [43], and in colorectal cancer models in rats [44]. *Clostridia* are a significant class of Gram-positive, often anaerobic, and spore-forming bacteria, known for producing the highest number of life-threatening toxins of any genus [45]. The genus *Clostridium sensu stricto 1* includes pathogenic bacteria such as *C. botulinum*, *C. tetani*, *C. chavoei*, and *C. perfringens*; they can produce various enterotoxins that affect the gut, such as *C. difficile* toxins A and B, histotoxins affecting soft tissue such as *C. perfringens* and *C. septicum* alpha-toxins, and neurotoxins affecting nervous tissue such as tetanus (*C. tetani*) and botulinum (*C. botulinum*) toxins [45]. Diseases range from gastroenteritis to abdominal disorders, colitis, muscle necrosis, soft tissue infections, tetanus, and botulism, among others. It was reported that 40% of patients with *Clostridium* infections are affected by colorectal cancer [44]. In such patients, the acidic, hypoxic environment created by anaerobic glycolysis of the cancer cells promotes spore germination of *C. septicum*, with subsequent infection [46]. *C. perfringens* infection induces oxidative stress, inflammation, and severe pathological changes in the intestinal mucosa [47]. Faecal microbiota transplantation in colorectal cancer mice reduced the abundance of this genus and reversed intestinal dysbiosis [44]. This ability to produce several types of toxins may explain its impact on the instability of the bacterial population in the gut and, consequently, its potential to increase DNA mutations, contributing to neoplasm development [44]. Hence, being increased in the MCT dogs, the genus *Clostridium* may contribute to the development of the disease.

The genus *Faecalibacterium*, a known producer of SCFA, such as propionate [48], was also reduced in dogs with MCT. Loss of SCFA-producing bacteria can disrupt the mucosal barrier and promote microbe-mediated host immune dysregulation, perpetuating a pro-inflammatory state and contributing to carcinogenesis [48]. Studies have also shown a lower abundance of *Faecalibacterium* spp. in dogs with IBD compared with healthy control dogs [49]. This genus is considered a crucial immune-modulatory bacterial group, and one of its bacterial species, *Faecalibacterium prausnitzii*, has also been found to be consistently reduced in human IBD patients, thus being considered an important bacterial taxon for maintaining microbial homeostasis [17].

Although not statistically significant, we found an increase in the *Streptococcus* genus community in diseased animals. This genus has also been amplified in the faeces of human patients with colorectal [50] and gastric [51] cancers, where it is considered an important cancer biomarker. Similarly, the genus *Blautia*, an anaerobic bacterium, was higher in the control animals. Ye et al. [52] explored the involvement of microbial signatures in chronic stress-induced breast cancer and found that reduced abundances of *Blautia* and its metabolite acetate may be associated with the carcinogenesis process. They also found that treatment with *Blautia* and acetate stimulated the antineoplastic responses of CD8^+^ T cells and reversed disease progression in female mice, concluding that *Blautia*-derived acetate may be the key to modulating immune responses to breast cancer. In fact, this genus was depleted in our group of cancer dogs. The probiotic effects of this genus, such as its ability to regulate host health and alleviate metabolic syndrome, have also been reported [53].

Some limitations of our study include the different home environments and commercial diets of dogs, which may act as confusing factors in microbial analysis, as well as a significant difference in average age between the two groups (mean age 4.6 in healthy animals vs. 8.9 years in MCT dogs). Since oncological diseases are more prevalent in older animals, the study group had a higher average age. Assembling a healthy, aged group of animals free of underlying pathologies and not receiving medication was challenging. Nevertheless, previous research suggests that disease is a major driver of microbiota differences than factors such as age, diet, or body weight [49]. Furthermore, 16S rRNA sequencing, while widely used in microbiota studies, has known limitations that should be considered: its resolution is restricted to the genus level due to the limited number of sequenced base pairs (300 bp), which does not cover the entire gene, thus reducing the ability to accurately characterise microbial communities. Moreover, this approach does not capture fungal or viral components of the microbiome, which are increasingly recognised as potentially significant contributors to cancer development and progression. Future studies incorporating metagenomics or multi-omics approaches could provide a more comprehensive understanding of the microbiome’s role in oncogenesis by including these often-overlooked microbial constituents. Therefore, more advanced techniques, such as metagenomics, are needed to achieve a more accurate and detailed understanding of microbiome composition and its complex interactions with the host.

## 5. Conclusions

In summary, this study presents the first characterisation of the faecal microbiota in dogs with MCT. We identified patterns of gastrointestinal dysbiosis similar to those described in other canine and human cancer patients. The faecal microbial communities of dogs with MCT differed significantly from those of healthy dogs. In further studies, we aim to evaluate the impact of these microbiota changes on disease progression, response to cancer treatments, and patient survival, contributing to the identification of new potential diagnostic, prognostic, and therapeutic biomarkers.

## Figures and Tables

**Table 1:** Distribution of breeds, sex, and age (years) of healthy control dogs.

Dog ID	Breed	Sex	Age (Years)
1	Mixed Breed	F	3
2	Portuguese Water Dog	F	6
3	Labrador Retriever	M	1
4	Mixed Breed	M	4
5	Mixed Breed	M	3
6	Yorkshire Terrier	M	8
7	Labrador Retriever	F	1
8	Pinscher	F	4
9	West Highland White Terrier	F	8
10	Mixed Breed	F	7
11	Mixed Breed	F	3
12	Mixed Breed	F	7
13	Pinscher	F	5
15	Schnauzer	M	2
16	German Shepherd	M	2
17	Vizsla	M	1
18	Mixed Breed	M	1
20	Labrador Retriever	M	2
21	Mixed Breed	F	8
22	Beagle	M	2
23	Mountain Cur	F	10
24	Beagle	F	5
25	Mixed Breed	F	5
26	Labrador Retriever	M	7
27	Mixed Breed	F	1
29	Pekingese	F	9
30	Cocker Spaniel	F	10
32	Portuguese Fila Dog	F	4

M—Male; F—Female.

**Table 2:** Distribution of breed, sex, age (years), and histopathological grading classification (Patnaik and Kiupel) of MCT-affected dogs.

Dog ID	Breed	Sex	Age (Years)	Grade (Patnaik)	Grade (Kiupel)	Tumour Size	Metastasis
1	Mixed Breed	F	11	III	High	≤3	No
2	Mixed Breed	F	9	II	Low	≤3	No
3	Labrador Retriever	M	10	II	High	≤3	Yes
4	Beagle	M	6	Chemotherapy	-	≤3	No
5	Labrador Retriever	F	9	II	Low	≤3	No
6	Boxer	M	9	I	Low	≤3	No
7	Boxer	M	8	II	Low	≤3	No
8	Mixed Breed	F	12	Chemotherapy	-	>3	No
9	Labrador Retriever	M	9	Subcutaneous	-	≤3	No
11	Labrador Retriever	F	12	II	Low	≤3	No
12	Mixed Breed	M	9	II	Low	≤3	No
13	Labrador Retriever	M	8	II	Low	≤3	No
14	Mixed Breed	M	7	Subcutaneous	-	≤3	No
15	Labrador Retriever	M	8	II	Low	≤3	No
16	Mixed Breed	F	4	II	Low	≤3	No
17	Labrador Retriever	F	9	Subcutaneous	-	≤3	No
18	Labrador Retriever	M	10	II	Low	≤3	No
19	Labrador Retriever	M	10	II	Low	>3	No
20	Mixed Breed	M	10	-	-	≤3	No
21	Golden Retriever	F	8	II	Low	≤3	No
22	Boxer	F	4	Subcutaneous	-	≤3	No
24	Golden Retriever	M	11	III	Low	≤3	No
25	Mixed Breed	F	11	Subcutaneous	-	≤3	No
26	Shar-pei	M	7	Chemotherapy	-	>3	No
27	Mixed Breed	F	15	Chemotherapy	-	>3	Yes
29	Labrador Retriever	F	7	II	Low	>3	Yes
30	Basset Hound	M	8	II	Low	≤3	No
32	Labrador Retriever	M	9	II	Low	≤3	No

M—Male; F—Female; Chemotherapy—these animals received chemotherapy as their first treatment (before tumour resection, which prevented histopathological grade classification—therefore, not applicable.

## Data Availability

Dataset available on request from the authors.

## Supplementary Materials

The following supporting information can be downloaded at: https://www.mdpi.com/article/10.3390/ani15152208/s1, Table S1: ASVs found in faecal samples of healthy dogs (CTR) and dogs with MCT (TUM); Table S2: Most abundant genera found in faecal samples of healthy dogs (CTR) and dogs with MCT (TUM); Table S3: Mean relative frequence of differentially abundant taxa (phylum, class, order, family, genus) in faecal samples of healthy dogs (CTR) and dogs with MCT (TUM); Table S4: Shared and unique genera found in faecal samples of healthy dogs (CTR) and dogs with MCT (TUM).
